# The Ethical Digital Surgeon

**DOI:** 10.2196/25849

**Published:** 2021-07-05

**Authors:** Kyle Lam, Sanjay Purkayastha, James M Kinross

**Affiliations:** 1 Department of Surgery and Cancer Imperial College London London United Kingdom

**Keywords:** digital surgery, ethics, data governance, robotics, digital surgeons, surgery, digital health care, smartphone app

## Abstract

This viewpoint explores the ethical and regulatory consequences of the digital transformation of the operating room. Surgical robotics is undergoing significant change and future advances will center around the capture and use of data. The consequences of creating this surgical data pipeline must be understood and digital surgical systems must prioritize the safeguarding of patient data. Moreover, data protection laws and frameworks must adapt to the changing nature of surgical data. Finally, digital surgeons must understand changing data legislation and best practice on data governance to act as guardians not only for their own but also for their patients’ data.

## Digital Surgery: Overview

Robotic surgery is currently undergoing significant change. Global medical device companies are entering a rapidly growing market based on economic optimism and a shifting clinical evidence base. COVID-19 has dramatically sped up this transformation with rapid global uptake in numerous digital platforms across health care [1]. The majority of these robots are systems built on incremental mechanical innovations that require a surgeon to operate a console that drives single, multiport, or endoscopic robotic arms. However, the real revolution is happening in the data that will be captured from sensors and systems embedded within these robotic platforms and streamed to the cloud. “Digital surgery” is defined as the capture, storage, analysis, and visualization of surgical data sets for precision surgery. It incorporates numerous enabling technologies such as artificial intelligence (AI), cloud computing and image augmentation, such as the use of 3D augmented reality guidance for the removal of complex tumors [2]. The hope is that digital robotic systems can be leveraged for real-time decision support surgery and it is this that will finally deliver the quantum leap in safety and clinical outcomes that robotic surgery has to date not yet provided. This compelling vision has created significant hype and general surgery robotic adoption rates are rapidly climbing [3-5].

## Ethical and Regulatory Challenges of Digital Surgery

What is far less clear are the ethical and regulatory consequences of suddenly turning on a limitless surgical data pipeline. Operating room data sets are unique, large, and heterogenous (eg, imaging, video, sensor data, text, team, and instrument performance) and each contains a priceless data surplus that will be mined for as yet unimagined purposes. It is unclear how patients undergoing surgery in the future operating room will be protected from data misuse or how hospital providers and patients will be reimbursed by the companies that use them to create their digital products.

However, these innovations do not just create ethical challenges for the patient. The future surgeon will also operate in a world where every minor action and decision will be subject to scrutiny. This moves beyond the capture of relatively blunt operative performance measures such as time, conversion rates, patient outcomes, and theater utilization into a paradigm of machine learning of an individuals’ total surgical performance. “Digital surgeons” who embrace this world will have much to gain through faster and safer learning curves, continuous education, and the delivery of safer procedures. Although digital surgeons will probably remain accountable for the decisions that they make, it is now possible that they will also have to contend with automation bias, opaque algorithms, and a rapidly evolving ecosystem of sophisticated cloud-based platforms and connected hardware. This will in turn create new challenges for consent and litigation, which are as of yet untested. The digital surgeon will serve as a guardian not only for their own data, but also for their patient’s data. It is therefore imperative that they understand and keep up to date with data legislation and best practice on data governance. “Binary surgeons” who do not have access to digital robotic platforms or who choose to reject them will be left in a perilous position where their performance will be compared with digitally augmented colleagues regardless of whether they “opt out.”

## “Streams”—Lessons Learned?

There is now a critical need for the surgical community to address the ethical and regulatory challenges of this brave new world. We can heed many lessons from the introduction of similar technologies in the public clinical domain. In July 2015, clinicians from the Royal Free London NHS Foundation Trust approached Google DeepMind Technologies Limited in order to create a smartphone app called “Streams” to aid clinicians in the management of acute kidney injury. The company was given access to the complete health records of all 1.6 million patients attending Barnet, Chase Farm, and Royal Free Hospitals over more than a 5-year period. Neither DeepMind nor the Royal Free obtained the requisite approval from the Information Commissioner’s Office, the Health Research Authority, or the Confidentiality Advisory Group [6]. In 2017, the Information Commissioner’s Office found several shortcomings in how data were handled, ordering an independent audit into “Streams” to be conducted [7]. While the audit concluded that the Royal Free and DeepMind’s actions were not unlawful [8], the episode demonstrates that patients should be aware of when and why their data are being used and have the ability to opt out if desired. The UK’s National Data Opt-Out, introduced in May 2018 [9], which allows patients to opt out of their confidential patient information being used for research and planning, is a key step in the right direction in this regard.

## Future Challenges

However, this may be more challenging in an operative environment. For example, if digital surgery systems are inherently dependent on data capture and analysis to function through networked robotic systems, what are the consequences for patients or surgeons who refuse or do not wish to share their data? National bodies such as the UK’s NHSX, responsible for the digitization of the National Health Service, have already established their national center of expertise overseeing data sharing agreements with industry [10]. The hope is that these organizations can create frameworks for delivering standardized guidance and contracts to support successful relationships between health care and industry in an equitable manner.

Irrespective of these initiatives, both patients and surgeons must have total oversight of what digital surgery companies are doing with our data and future algorithm development. US and EU regulators are currently trying to ensure that artificial intelligent systems are safely introduced into the operating room. Data protection laws such as the EU’s General Data Protection Regulation (GDPR) and the USA’s Health Insurance Portability and Accountability Act (HIPAA) also provide a framework for data protection. It is questionable whether these frameworks do enough to protect surgical patients; the “surgical data surplus” is priceless and health care organizations must guard this revenue source closely if we are to build sustainable digital surgical systems and keep control of the health inequality gap.

Studies have shown that health care providers bear the weight of public expectation when patients consent to share their data [11]. Digital surgeons must therefore have basic AI literacy and foundational knowledge in data governance and protection. Moreover, the digital surgeon must now lead in the creation of “trustworthy AI” which is legal, ethical, and fit for surgical purpose. In turn, future robotic systems must be developed, tested, and trialed in a learning system that incorporates digital surgery as a core pillar of its development. Finally, international surgical bodies must also determine what role they are going to play in this brave new world and how they are going to certify and train the digital surgeon of the future (Textbox 1).

**Key messages.**

- Digital surgical systems must prioritize the safeguarding of patient data.
- Data protection laws and frameworks must adapt to the changing nature of surgical data.
- Future surgeons must be artificial intelligence literate and understand data governance and protection.

## Abbreviations

AI: artificial intelligence
GDPR: General Data Protection Regulation
HIPAA: Health Insurance Portability and Accountability Act
